# *Corynebacterium matruchotii*: A Confirmed Calcifying Bacterium With a Potentially Important Role in the Supragingival Plaque

**DOI:** 10.3389/fmicb.2022.940643

**Published:** 2022-07-06

**Authors:** Qinyang Li, Fangjie Zhou, Zhifei Su, Yuqing Li, Jiyao Li

**Affiliations:** ^1^State Key Laboratory of Oral Diseases, National Clinical Research Center for Oral Diseases, West China Hospital of Stomatology, Sichuan University, Chengdu, China; ^2^Department of Cariology and Endodontics, West China Hospital of Stomatology, Sichuan University, Chengdu, China

**Keywords:** *Corynebacterium matruchotii*, supragingival plaque, biofilm structure, bacterial interaction, oral diseases

## Abstract

*Corynebacterium matruchotii* is a reported calcifying bacterium that can usually be isolated from dental calculus and induce mineralization *in vitro*. In recent years, based on *in situ* hybridization probe and sequencing technology, researchers have discovered the central “pillar” role of *C. matruchotii* in supragingival plaque, and many studies focused on bacterial interactions in the biofilm structure dominated by *C. matruchotii* have been conducted. Besides, *C. matruchotii* seems to be an indicator of “caries-free” oral status according to imaging and sequencing studies. Therefore, in this review, we summarize *C. matruchotii* ‘s role in supragingival plaque based on the structure, interactions, and potential connections with oral diseases.

## Introduction

*Corynebacterium matruchotii* (previously known as *Leptothrix buccalis* and *Bacterionema matruchotii*) is a gram-positive aerobic bacterium that was first discovered in 1925 (Ennever and Creamer, 1967). Of all the anatomical sites in the oral cavity, it occurs most in dental plaque on the tooth surface and serves as one of the most predominant bacteria at the site (Mark Welch et al., 2016; Eriksson et al., 2017; Esberg et al., 2020). During the 1960's−2000's, the bacterium was verified to precipitate calcium phosphate on the intracytoplasmic membranes and share a similar calcified nucleator with dental calculus (Vogel and Smith, 1976; Boyan-Salyers et al., 1978; Ennever et al., 1978, 1979; Sidaway, 1978a). However, after these investigations reported this calcifying ability, studies on *C. matruchotii* in the oral ecosystem were strikingly absent from the literature for the next decade or so.

In the past few years, with the development of laboratory techniques such as fluorescence *in situ* hybridization with probes and high-throughput sequencing, the taxonomy of bacteria in highly structured dental plaque has been clearly depicted, and *C. matruchotii* has once again become a focus of research. As reported recently, *C. matruchotii* is in the center of supragingival plaque and interacts with a variety of bacteria around it *via* its special morphology, which may indicate a new sight for plaque development and maturation. In addition, several studies on oral diseases associated with supragingival plaque have pointed out *C. matruchotii* as a potential indicator of healthy status since there is an increase in the relative abundance of *C. matruchotii* compared with the morbid state, implying *C. matruchotii* might play more hidden roles in the development of different oral status.

This review is aimed at summarizing the latest findings of *C. matruchotii* in supragingival plaque, including its bio-geographic position in the organized oral biofilm, interactions with other bacteria, and possible effects in oral diseases, attempting to better understand the characteristics of dental plaque in various conditions to explore ways to maintain oral health from a microbial perspective.

## *C. matruchotii* in the Structure of Supragingival Plaque

*C. matruchotii* was discovered in much higher abundance and prevalence in the supragingival plaque and subgingival plaque than in saliva or other oral surfaces such as the tongue, buccal mucosa, and keratinized gingivae (Mark Welch et al., 2016). Therefore, the potential big role for it in oral biofilms came to attention. In 2016, Borisy et al. used a fluorescence *in situ* hybridization probe to determine the bio-geographic location of each bacterium and first clearly showed the central position of *C. matruchotii* in the hedgehog structures in healthy people's supragingival plaque (Mark Welch et al., 2016; Borisy and Valm, 2021). The “hedgehog” structure is a special consortium of various bacteria named by its morphology seen under electron microscopy as spiny, radially oriented filaments, and within the structure, *C. matruchotii* stood in the center of the field of vision with a variety of bacteria surrounding it. Combined with a recent metaproteome study claiming that *C. matruchotii* represented a large proportion of the protein activity in supragingival dental plaque, which suggested its strong biological activity and metabolic capacity (Belda-Ferre et al., 2015), a hypothesis can be drawn that it undertakes numerous information exchanges among bacteria and performs important functions in healthy oral status. Additionally, based on the recent research conducted on the metabolic current production by *C. matruchotii*, the radial “hedgehog” structure may support electrically coupled organics oxidation and oxygen reduction in dental biofilm as in the cases of long-range extracellular electron transport which could facilitate colonization of various bacteria in the anaerobic environment (Naradasu et al., 2020). From the basal layer closest to the tooth surface outward, *Corynebacterium* traversed through the whole distance; from the staining, an ordered arrangement could be seen in the form of a small amount of *Actinomyces* located near the tooth base, *Capnocytophaga/ Fusobacterium/ Leptotrichia* forming a ring between the periphery and the base, *Neisseriaceae* clustering in and near the periphery, and *Streptococcus/ Haemophilus/ Porphyromonas* being located at the periphery encircling *Corynebacterium* as the “corncob structure.” The organized “hedgehog” structure was determined by the metabolism and biochemicals of different bacteria according to the group supposed, and *Streptococcus* seemed to be the major driver of this organized structure which used external oxygen to produce hydrogen peroxide, carbon dioxide, and a series of metabolites to impact growth and survival adaption of other bacteria.

## The Interactions Between *C. matruchotii* and Other Commensal Bacteria Close in Bio-Geography to Supragingival Plaque

The development and maturation of oral biofilm were determined by the relationship between participating bacteria, which presented as bacterial co-aggregation and co-adhesion (Kolenbrander et al., 2010). Recently, to further explore a vital part of *C. matruchotii* in biofilm, Anders Esberg et al. systematically performed an *in vivo* and *in vitro* study to seek bacterial interactions in coaggregation and co-adhesion assays (Esberg et al., 2020). Consistent with the fact that *C. matruchotii* were in close proximity with *Actinomyces spp* reported by Borisy, Esberg et al. demonstrated an indispensable role of *Actinomyces spp* for *C. matruchotii* binding on the tooth surface. The group hypothesized that *Actinomyces naeslundii* could be an initial tooth colonizer binding to the saliva pellicle and providing attachment sites for *C. matruchotii* since *in vivo* the abundance of *A. naeslundii* was similar to *C. matruchotii* at all ages, and *in vitro, A.naeslundii* bound *C.matruchotii* both in the state of plankton and biofilm and had the ability to recruit *C. matruchotii* to the tooth surface.

In the “hedgehog” consortium of supragingival plaque, a unique structure named “corncob” could be seen at the periphery of supragingival plaque away from the enamel surface (Mark Welch et al., 2016), which was formed at the very beginning of the acquired pellicle and closest to the outside air, which determined a different metabolism to the inside bacteria (Rickard et al., 2003). This local, specific structure was made of the central filament *C. matruchotii* encased by cocci *Streptococcus*, which could not be formed around other filamentous bacteria nearby such as *Fusobacterium, Leptotrichia*, or *Capnocytophaga*, revealing a particularly close relationship between *C. matruchotii* and *Streptococcus*. Previous studies showed that the species belonging to *Streptococcus* involved in corncob structure included *Streptococcus sanguis* and *Streptococcus cristatus* (Listgarten et al., 1973; Takazoe et al., 1978). However, the results claimed by Anders Esberg et al. only verified the co-aggregation and co-adhesion between *C. matruchotii* and *Streptococcus cristatus*, which may imply a connection that did not result from directly binding specific components to the respective cell surfaces. Puthayalai Treerat et al. co-cultured *Corynebacterium* and *S. sanguinis* in transwell culture plates that prevented direct contact between bacteria and found out free fatty acid transported by membrane vesicles of *Corynebacterium* could elongate chains and promote fitness of *S. sanguinis* (Treerat et al., 2020). The free fatty acids mediated by membrane vesicles as potential signaling molecules to impact *Streptococcus* and participate in biological activity without direct contact were also reported in other *Corynebacterium* (Boyan et al., 1984; Bomar et al., 2016), which implied a specific role of membrane vesicles enveloping fatty acids.

Besides, around the “corncob” region, an interesting manifestation could be observed: only when *Streptococcus* bound to *Corynebacterium* did *Haemophilus/Aggregatibacter* appear next to *Corynebacterium* (Mark Welch et al., 2016). Recently, a group verified the co-occurrence between *Haemophilus parainfluenzae* and *Streptococcus mitis* and assumed their proximity may be attributed to *H.parainfluenzae*'s dependence on NAD produced by *S. mitis* (Perera et al., 2022). Though many *streptococci* shared the common ability to generate hydrogen peroxide (H_2_O_2_), which inhibited other bacteria's growth, *H.parainfluenzae* seemed to evolve a hydrogen peroxide redundant system on account of frequent co-occurrence with *Streptococcus* (Kreth et al., 2009; Perera et al., 2022). Thus, from the local “corncob” region, it can be confirmed that behind the organized architecture of supragingival plaque, bacteria do have functional connections that match spatial structures, and there may be a strict temporal order for them to be involved. Additionally, *C. matruchotii*, as the predominant bacteria of *Corynebacterium* in the supragingival plaque, probably plays a big part in the beginning in shaping the biofilm structure.

## *C. matruchotii* in Oral Diseases Associated With Supragingival Plaque

### *C. matruchotii* and Caries

**Figure 1:**
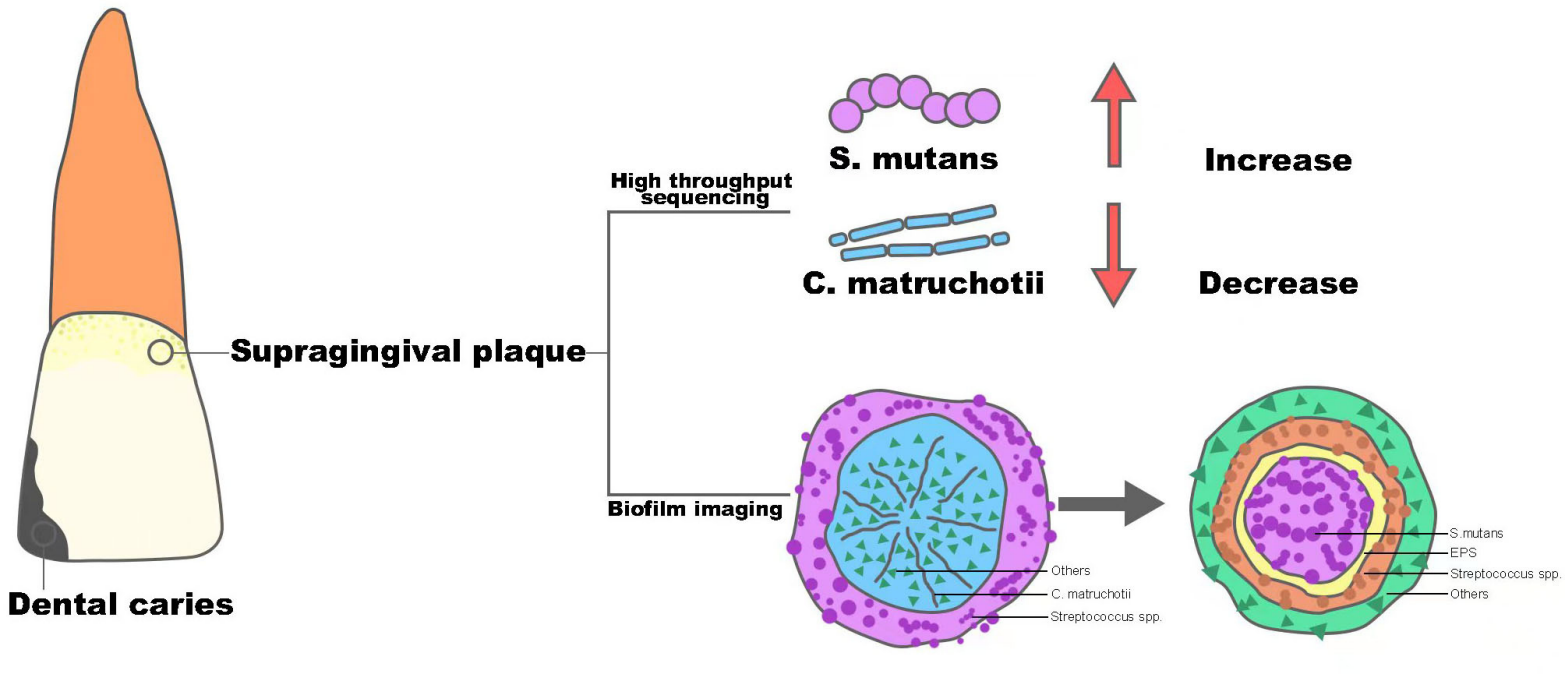
The structure alteration of supragingival plaque from healthy oral status to dental caries.

Dental caries remains one of the most prevalent chronic diseases in both children and adults, and it is shown to be a result of the demineralization of tooth enamel by acid corrosion (Heng, 2016). *Streptococcus mutans* is the primary pathogen of dental caries, and based on its strong ability of acid production and acid resistance, along with its possession of glycotransferase, it can use dietary sugar to produce insoluble glucans to form oral biofilm and generate acid to decalcify dental enamel and cause caries (Johansson et al., 2016). Recently, with the popularity of sequencing technology in clinical practice, the role of *C. matruchotii* seems to act as an opposite to *S. mutans* and tends to be considered as a marker of a caries-free state, which is depicted in Figure 1.

Muawia A Qudeimat et al. collected supragingival plaque samples from 64 caries-active and 64 caries-free middle eastern children and performed 16S rRNA sequencing (Qudeimat et al., 2021), and their results showed that *C. matruchotii* was relatively more abundant in the caries-free group. This result was consistent with the research conducted by Nezar Noor Al-Hebshi et al. using metagenome sequencing (Al-Hebshi et al., 2019). Based on the abundance alteration in different situations, these findings implied that *C. matruchotii* may own protective property and could be a representative species for caries-free status. At the same time, in all these studies, *S. mutans* showed a predominant abundance in the caries group and with the progression of caries, the abundance of *S.mutans* became higher, while the abundance of *C. matruchotii* decreased gradually (Gross et al., 2010). The consequence seemed to show a reciprocal relationship between *S. mutans* and *C. matruchotii* in microecology of supragingival plaque and dental caries was the outcome of transferred superiority from *C. matruchotii* to *S.mutans*.

In 2020, Dongyeop Kim et al. discovered a 3D corona-like structure with a high frequency of detection in people with caries by fluorescence *in situ* hybridization in intact biofilms formed on carious teeth of toddlers; this was constructed with an inner core of *S. mutans* surrounded by outer layers of other bacteria linked via extracellular polysaccharide (Kim et al., 2020). The highly organized rotund-shaped architecture, on the one hand, created localized regions of acidic pH and caused acute enamel demineralization, on the other hand, built a protective barrier against antimicrobials while increasing bacterial acid fitness. Compared with the *in situ* biofilm imaging study in healthy people by Gary G. Borisy et.al, the detection rate of “hedgehog” centered on *C. matruchotii* was cut in half, however, at the same time, the dominant “rotund-shaped” *S. mutans* became the most commonly detected architecture in people with caries (Mark Welch et al., 2016). Thus, combined with the “pillar” role of *C. matruchotii* in the structure of dental plaque in healthy people, it could be inferred that *C. matruchotii* might perform a key part in the formation and stabilization of healthy dental plaque. In contrast, the reduction of *C. matruchotii* may directly lead to the break of biofilm homostasis. Meanwhile, the decreased abundance of *C. matruchotii*, based on the close relationship shown in corncob structure of supragingival biofilm, might also be part of the reduction of commensal *streptococci*, such as *Streptococcus gordonii* and *S. sanguinis* (Agnello et al., 2017; Richards et al., 2017), reported before, which showed an antagonistic effect against *S. mutans via* their capability of producing hydrogen peroxide (Kreth et al., 2008). The alteration could finally contribute to a replacement of dominant species in the oral ecosystem and induce the formation of diseased-adapted supragingival plaque determined by *S. mutans* that lead to disorders in acid-base metabolism and eventually cause the demineralization of tooth enamel. In general, *C. matruchotii* is a potential indicator of a caries-free state, and the reduction of its abundance may indicate the evolution from healthy plaque to caries biofilm.

### *C. matruchotii* and Supragingival Calculus Associated With Periodontal Diseases

**Figure 2:**
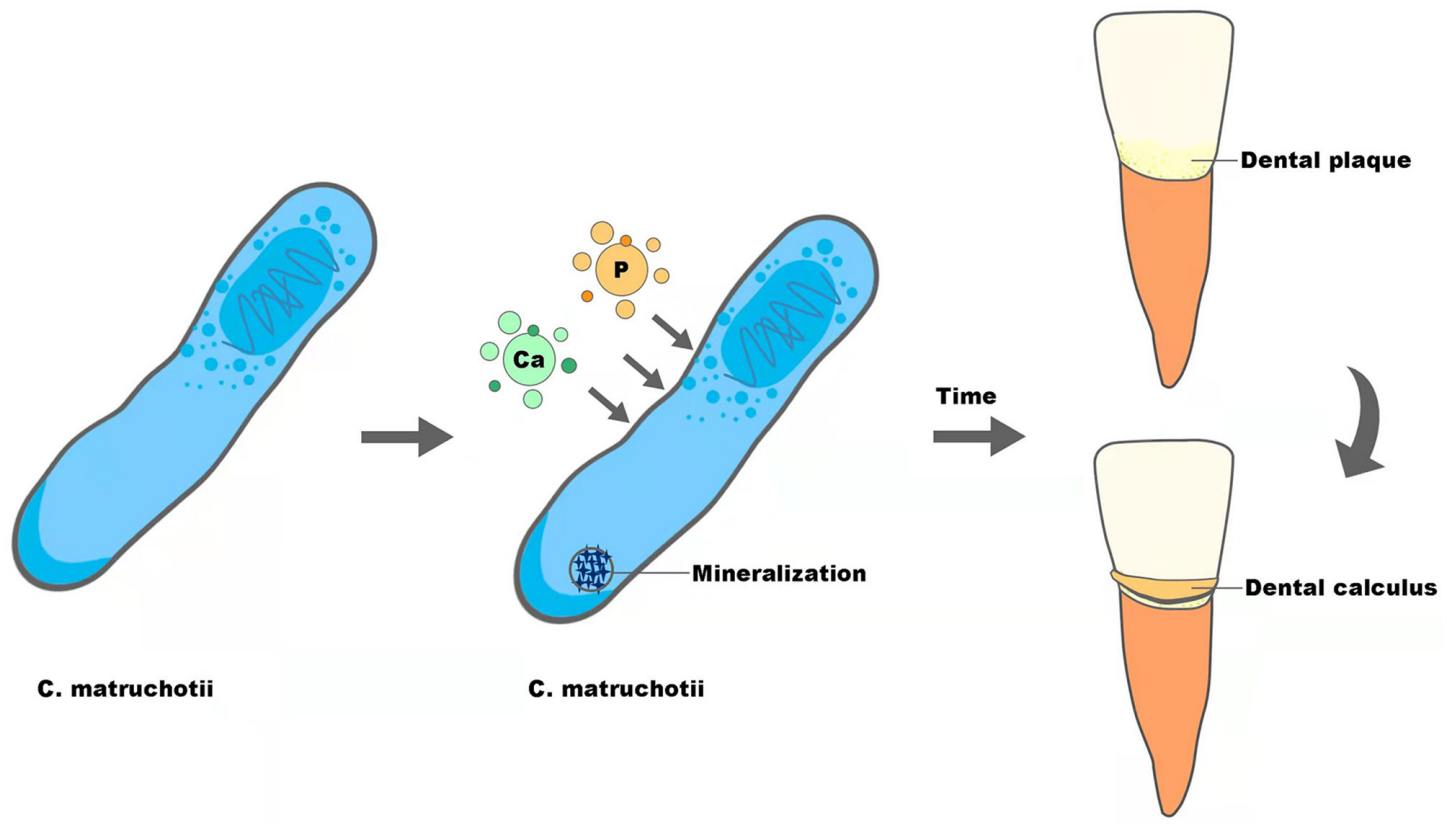
The potential role of *C. matruchotii* in the formation of dental calculus.

Supragingival calculus is the plaque and sediment that has calcified or is calcifying on the tooth surface touching or above the gingival margin, which can cause gingival inflammation and contribute to periodontal diseases. As reported before, *C. matruchotii* was a calcified bacterium that had the strong ability to deposit calcium and induce mineralization *in vitro* and may participate in the formation of supragingival calculus (Sidaway, 1978a; Takazoe and Itoyama, 1980; van Dijk et al., 1998) (Figure 2). With the superior ability to accumulate and mineralize calcium (Sidaway, 1978b), together with the fact that salivary levels of calcium increased in higher calculus formers and in periodontal diseases (Mandel, 1972; Sutej et al., 2012), *C. matruchotii* seemed to be an “organ” responsible for calcium metabolism in oral ecosystems analogous to *Entotheonella sp* in marine ecosystems in that they may prompt a similar absorption, accumulation, and mineralization process of ions (Keren et al., 2017).

The reason for supragingival calculus to promote periodontal diseases, as past scholars have highlighted, is attributed to the porous structure derived from dental calculus, which accommodated a large number of periodontitis-related pathogenic bacteria such as *Porphyromonas gingivalis* and their virulence factors (Socransky, 1970; Velsko et al., 2019). Recently, in addition to bacterial factors, studies showed that crystal properties or small calcium phosphate particles contained in dental calculus could directly cause periodontal inflammation as well. Experiments by Jorge Luis Montenegro Raudales et al. demonstrated that after heating dental calculus at 250°C for 1 h to eliminate residual bacteria, the treated calculus still activated NLRP3 inflammasome and promoted IL-1β secretion, which was involved in bone resorption and could also be induced by *P. gingivalis* (Ziauddin et al., 2018). In another study, Yu Sakai et al. found that tiny calcium phosphate particles stimulated gingival epithelial cells through the NF-kB pathway leading to a significant increase in the expression of interleukin-8 at the gene and protein levels, which could attract more neutrophils to participate in the development of periodontitis (Sakai et al., 2014). Thus, whether supragingival calculus is a deposit of pathogenic bacteria or an independent physical stimulus factor, the potentially important calculus former, *C. matruchotii*, seems to indirectly influence the progression of periodontal diseases.

The formation of dental calculus can be separated into three steps, formation of the acquired pellicle, plaque maturation, and plaque mineralization, and the first two are similar to the formation of dental biofilm. Inspired by recent research displaying dental calculus as the revelator of information about plenty of biological information (Weyrich et al., 2017; Jersie-Christensen et al., 2018; Willmann et al., 2018), we speculate that calculus could also be able to mirror the characteristics of dental plaque that forms it. The ability of calcification, along with the relatively high abundance and biogeography in the center of supragingival plaque, may give *C. matruchotii* a necessary role in calculus formation, though the transformation from dental plaque to calculus indicates alteration of plaque composition and structure (Velsko et al., 2019). Therefore, to give a more convincing conclusion about the role of *C. matruchotii* in supragingival calculus, studies with more samples are needed.

## Conclusion and Future Perspectives

After a long period of silence, *C. matruchotii* has once again appeared in research as a “pillar-like” structure in supragingival plaque, studies declaring its important role in oral biofilm and close interaction with numerous resident oral bacteria. Thus, it is promising to explore cooperative or competitive relationships between *C. matruchotii* and other commensals for a better understanding of the mechanisms behind the unique structure. Moreover, since interactions among bacteria determine the order for different bacteria joining in the formation of plaque, dynamics studies on a timeline can help us have a greater knowledge of the formation process of supragingival plaque and discover the “keystone” to stabilize or facilitate healthy plaque. The distinctive supragingival architecture along with microbial high-throughput sequencing results between caries-active and caries-free people shows the potential of *C. matruchotii* as an indicator of a healthy state, so it's worthy to investigate the function of the structure dominated by *C. matruchotii*. Additionally, it is also intriguing to see how carious “corona” replaces the healthy “hedgehog” to seek essential external or internal factors that cause a change in the nature of plaque; this can provide more insights into the development of strategies to prevent caries, precisely targeting on specific biofilm architecture.

Dental calculus in contact with gingival epithelial can induce inflammation to some extent and be an important contributing factor to periodontal diseases. The hidden reasons for its pathogenicity lie in the pathogens stuck in it as previously demonstrated and crystalline substances as recently claimed. *C. matruchotii* is confirmed to have a calcified ability, and it serves a central role in supragingival plaque establishing close associations with plenty of bacteria, which endow it with a non-negligible effect in the formation of biofilm and calculus. Moreover, in subgingival plaque of patients with periodontitis, *C. matruchotii* was reported to over-express a large number of putative virulence factors that could have importance in the evolution of the disease (Duran-Pinedo et al., 2014). Therefore, it is interesting to conduct studies on the relationship between *C. matruchotii*, calculus, and periodontal diseases, which may help us better understand the role *C. matruchotii* plays in plaque-related gingival diseases. Moreover, combined with the latest hot reports pointing out dental calculus as a potential repository of bio-information, we make an assumption that it can also reflect the bacterial composition of dental plaque in different calculus states, which may provide some clues on whether there are certain bacteria such as *C. matruchotii* that determine an individual's heterogeneity in the number and rate of calculus formation.

Apart from dental caries and periodontal diseases, there has been a study conducted on the bacteria isolated from patients with apical periodontitis demonstrating that *C. matruchotii* was present significantly more frequently in the root canal of patients with chronic periapical periodontitis compared with patients with endo-perio lesions or pulp necrosis without obvious changes in the periapical tissue X-ray image (Korona-Glowniak et al., 2021). Apical periodontitis is a result of pulpitis, and the bacteria probably contributing to apical inflammation came from the carious lesion, which went through the apical openings of the root canals, the lateral or chamber-periodontium canals, and the pathological gingival pocket. *C. matruchotii* has hardly been investigated in this apical niche, and the abnormal enrichment in this place may make its role distinct from that in dental biofilm. Besides, *C. matruchotii* was also found a higher prevalence and a dominant part in saliva and buccal mucosa of patients with oral lichen planus, a common chronic mucocutaneous inflammatory disease (Zhong et al., 2020). To sum up, *C. matruchotii* may perform multiple roles in different areas of the oral cavity and thus deserves more studies in the future.

## Author Contributions

QL, FZ, YL, and JL: conceptualization, methodology, and writing-original draft preparation. QL, FZ, ZS, YL, and JL: writing-review and editing. YL and JL: supervision and project administration. All authors contributed to the article and approved the submitted version.

## Funding

This study was supported by the National Natural Science Foundation of China (81991500, 81991501).

## Conflict of Interest

The authors declare that the research was conducted in the absence of any commercial or financial relationships that could be construed as a potential conflict of interest.

## Publisher's Note

All claims expressed in this article are solely those of the authors and do not necessarily represent those of their affiliated organizations, or those of the publisher, the editors and the reviewers. Any product that may be evaluated in this article, or claim that may be made by its manufacturer, is not guaranteed or endorsed by the publisher.
